# Hesperidin loaded bilosomes mitigate the nephrotoxicity induced by methotrexate; biochemical and molecular in vivo investigations

**DOI:** 10.1186/s12882-025-04328-4

**Published:** 2025-07-21

**Authors:** Shiemaa H. Mahmoud, Walaa A. Moselhy, Ahmed F. Azmy, Fatma I. Abo El-Ela

**Affiliations:** 1 Biotechnology and Life Sciences Department, Faculty of Postgraduate Studies for Advanced Sciences, Beni-Suef University, Beni-Suef, 62511 Egypt; 2 Department of Forensic Medicine and Toxicology, Faculty of Veterinary Medicine, Beni-Suef University, Beni-Suef, Egypt; 3 Department of Microbiology and Immunology, Faculty of Pharmacy, Beni- Suef University, Beni-Suef, 62511 Egypt; 4 Pharmacology Department, Faculty of Veterinary Medicine, Beni-Suef University, Beni-Suef, 62511 Egypt

**Keywords:** Hesperidin, Bilosomes, Nephrotoxicity, *Nrf2*, *Keap1*, *Bcl2*, *Bax*

## Abstract

**Background:**

Drugs, chemicals, and poisons may damage kidneys and cause chronic renal disease. About 20% of community and hospital acute renal failures are drug-related.

**Objectives:**

Hesperidin-loaded bilosomes (HES-BS) nanoformula was tested for nephroprotection against methotrexate (MTX)-induced kidney injury in rats. Thin-film hydration produced HES-BS nanoformula. Drug-loading capacity, encapsulation efficiency (EE %), FTIR, DSC, zeta sizer, and potential were employed for characterization, coupled with an in vitro release study. In vivo pharmacological investigations on White male albino rats measured metabolic parameters, oxidative stress indicators, Nrf2 /Keap1 and BCL2/Bax gene expression, and histopathological alterations.

**Results:**

The HES-BS nanoformula was synthesized with 162 nm particles and − 21.6 mv potential charge. The Transmissions electron microscopy (TEM) showed spherical HES-BS. Hesperidin-excipient compatibility was shown by FTIR and DSC investigations on the modified formulation, with 89.1% EE%. In vitro drug release showed 56% release after eight hours and 60.1% after 24 h, with greater bioavailability than crude HES. The IC₅₀ value of hesperidin decreased from 264 µg/mL to 106.2 µg/mL upon bilosome loading in Vero cells. MTX’s nephrotoxicity was mitigated by the HES-BS nano formula’s effects on creatinine, urea, uric acid, eGFR, Na, and K levels. Malondialdehyde (MDA) and Nitric Oxide (NO) were notably decreased, whereas Glutathione (GSH) and Superoxide Dismutase (SOD) were markedly elevated. Also, *Nrf2* and *Bcl2* were upregulated, while *Keap1* and *Bax* were downregulated. Additionally, the produced nanoformula improved the histopathological function.

**Conclusion:**

Our novel HES-BS nanoformula had potent nephroprotective activity by reducing the toxic effects of MTX treatment by improving biochemical indicators of kidney function, oxidative stress markers, anti-apoptotic gene expression, and apoptotic gene expression, as well as histopathological improvement.

**Supplementary Information:**

The online version contains supplementary material available at 10.1186/s12882-025-04328-4.

## Introduction

Nephrotoxicity is the rapid deterioration of kidney function resulting from the toxic properties of medications and various other substances. It arises from various mechanisms, such as inflammation, toxicity to the renal tubules, crystal-induced kidney damage, and glomerular damage [1]. Methotrexate, a dihydrofolate reductase inhibitor, is prescribed to manage several malignant and non-malignant conditions. It could be crystallized and precipitated inside the renal tubules, resulting in crystalline nephropathy [2]. Moreover, MTX generates reactive oxygen species (ROS), lowers the efficacy of antioxidant enzymes, causes apoptosis, and damages both malignant and healthy cells, ultimately increasing the risk of renal impairment [3–5].

Several investigations have indicated that various naturally occurring compounds have medicinal value as an alternative source of drugs, with considerable advantages for the alleviation of MTX-induced kidney impairment [6–8]. Hesperidin (HES) is a phytoflavanone glycoside natural compound that offers several pharmacological properties, including anti-inflammatory, hepatoprotective, antidiabetic, hypolipidemic, antioxidant, and anti-carcinogenic action [9–12]. Considering the promising therapeutic effects, HES’s low water solubility and bioavailability have restricted its oral distribution [13]. Thus, improving the solubility of HES might assist in increasing its dissolution, stability, bioavailability, and biological effects [14].

Bilosomes (BS) are lipid nanovesicles loaded with bile salts with a high capacity to pass via biological membranes [15]. Integrating bile salts within the framework of BS has been found to minimize the breakdown of nanovesicles in the gastrointestinal tract (GIT), increase permeability, and make oral administration more efficient [16]. Furthermore, negatively charged bile salts involving sodium deoxycholate (SDC) were observed to improve the colloidal integrity of BS [15, 17]. Additionally, because of its narrow nano-size range and fluidizing activity, BS might provide effective permeability across biological barriers, accelerating medication absorption [18, 19].

To our knowledge, no prior research has been conducted on the preventive properties of the HES-BS nano formula versus MTX-induced nephrotoxicity. The current research aims to examine the efficacy of a novel formulation of HES-BS in overcoming MTX-induced nephrotoxicity via in vitro and in vivo experiments.

## Materials and methods

### Drugs and chemicals

Hesperidin, lecithin, sodium deoxycholate (SDC), ethanol, chloroform, and a dialysis bag of molecular weight cut off 12–14 kDa were bought from Sigma Chemicals Co., St. Louis, MO. Methotrexate was purchased from MYLAN SAS – France, imported by: RAMCO (Cairo, Egypt). The remaining compounds were of analytical quality and came from an ordinary commercial source.

### Preparation of Hesperidin-Loaded bilosomal vesicles

BS vesicles were generated utilizing several ways; in our study, the BS was made using the thin-film hydration process reported by Chen et al. but with some changes [20]. Lecithin (0.425gm) together with SDC (0.075 gm) and hesperidin (0.052 gm) were weighed into a round-bottom flask and liquified in chloroform: methanol 1:1, followed by sonication for 30 min and followed by removing solvents under a vacuum by adjusting the rotary evaporator instrument at 100 rpm (Rotavapor, Heidolph VV 2000, Germany), leading to a thin film within the flask. Subsequently, the acquired film was soaked with 5 ml of phosphate buffer saline (PBS) at a pH of 7.4. They were bath-sonicated for an hour to reduce the particle size of the produced vesicles. For further studies, the vesicular dispersion was utilized after being allowed to grow overnight at 4 °C. A preliminary stability assessment over a 3-month period at 4 ± 2 °C had been conducted.

### Characterization of hesperidin-bilosomes nano formula

#### Measurement of hydrodynamic size and surface charge of HES-BS nanoparticles

The dynamic light scattering (DLS) technique was used to evaluate the hydrodynamic size and zeta potential of the distributed HES-BS nanoparticles (ZS90 Zetasizer, Malvern, UK) [21]. The alteration of the zeta potential of the HES-BS nanoformula was measured using a dispersant that had a refractive index of 1.454 consisting of PBS (pH 7.4). Each measurement was performed in triplicate (*n* = 3).

#### High-resolution transmission electron microscope (HR-TEM) study

The HES-BS nanoformula was examined for its particle size and surface morphology using a high-resolution transmission electron microscope (HR-TEM) (JEOL, JEM-2100, Tokyo, Japan) worked at 200 kV. Firstly, the suspension of the prepared formula was sonicated for about 20 min on an ultrasonicator (Crest Ultrasonics Corp., New Jersey, USA).

Secondly, a few drops of the diluted HSB-BS nano formula were deposited on the surface of a carbon-coated copper grid and permitted to dry before the investigation [22].

#### Entrapment efficiency percentage (%EE)

To determine the entrapment effectiveness of the produced bilosome, the HES-BS nanoformula was centrifuged at 14,000 r.p.m and 4 °C for 45 min. The quantity of free hesperidin in the supernatant was measured at λ = 280 nm using a UV-VIS analyzer. Each measurement was executed three times (*n* = 3). The entrapment efficiency (EE %) was intended using the following equation [23]; EE (%) = [(Total amount of HES -Free amount of HES)/Total amount of HES] × 100.

#### Fourier transform infrared spectroscopy

FTIR can analyze interfaces to evaluate the surface adsorption of functional groups in nanoformula [24]. The FTIR spectra of the prepared samples were done using a Vertex 70 infrared spectrophotometer (Bruker, Karlsruhe, Germany). The dry sample and physical mixture (1 mg) were mixed well with a KBr powder (100 mg) and then squeezed into pellets. The spectra of the prepared samples were done using a wavenumber ranging from 400 to 4000 cm^− 1^ at 25 °C, a resolution of one cm^− 1^, and a gathering of 64 scans.

#### Differential scanning calorimetry (DSC)

The thermograms of crude hesperidin and HES-BS nanoformula samples were recorded using the DSC instrument (DSC 1 STAR system, Switzerland). The 5 mg of the lyophilized HES-BS and pure HES were kept distinctly in the pan. Then heated up to 300 ^o^C at a frequency of 10 ^o^C /min and N_2_ gas was pumped into the compartment at a rate of 20 mL/min [25].

#### In vitro release study of HES-BS nano formula

The amount of hesperidin released from the prepared HES-BS nano formula was evaluated using a modified dialysis bag method [26]. Aliquots of the nanodispersion (equivalent to 3 mg of HES) were introduced into a glass cylinder, which was strongly covered at one opening side with a presoaked dialysis membrane with a Mol. Wt. cut off = 12,000 Da. For establishing sink circumstances, 70 mL of PBS pH 7.4 served as a release medium. During the release experiments, the rotation speed was customized to 100 rpm at 37 °C.

A sample (2 mL) was obtained at planned periods of 1, 2, 4, 6, 8, and then 24 h, and they were replaced with fresh media of equivalent volume to justify a constant volume. The in vitro dispersion behavior of 3 mg of pure HES was also studied. Hesperidin concentration was determined using a spectrophotometer at λ max = 280 nm. Each measurement was done in triplicate.

### In vitro cytotoxicity assay and IC_50_

Yellow MTT, which is a tetrazole compound; 3-(4,5-Dimethylthiazol-2-yl)-2, 5-diphenyltetrazolium bromide, undergoes reduction to generate purple formazan specifically inside the mitochondria of viable cells. The decrease occurs only when mitochondrial reductase enzymes are functioning, therefore allowing for a direct correlation between conversion and the number of viable cells.

#### Cells and samples

The cytotoxicity test was performed on Vero Cell line cells (ATCC, USA). The cells were grown.

in DMEM media with 10% FBS, 100 units/mL penicillin, and 100 mg/mL streptomycin. Samples were kept at 37 °C in a moist environment containing 5% CO2. All samples were diluted in DMEM complete medium at 37 °C to produce a stock solution. Six to seven concentrations of two-fold serial dilutions were carried out and the started concentration was 100 µg/mL.

#### MTT assay

In summary, a layer of cells was cultivated on 96 well-microliter plates for 24 h. The cultivated cells were treated to varying concentrations of the test substances in triplicate and kept at 37 °C in a CO2 environment for 48 h. After carefully introducing 20 µL of a 5 mg/ml MTT, each well was incubated at 37 °C for four hours. Next, cautiously remove the medium and introduce 150 µl of isopropanol. Enclosed with aluminum foil, shake the cells on an orbital shaker for 15 min. Ultimately, the optical density (OD) was assessed at 570 nm via a microplate reader [27, 28]. IC50 was calculated using GraphPad Prism software (San Diego, CA, USA).

### In vivo study

#### Experimental animals and study design

We bought 4-week-old white male Wistar albino rats from the VACSERA in Egypt. They weighed between 150 and 240 g. During an initial week of acclimation, the rats were housed in special plastic cages at a constant temperature of 25 °C and a light-dark cycle of 12:12 h.

To produce nephrotoxicity in rats, a single intraperitoneal dose of methotrexate (20 mg/kg) was administered to each rat at the beginning of the study, on day 1 [29].

Twenty-four rats were randomly assigned to four groups; each group contained six rates and categorized as follow: control group (saline, 14 days), MTX- group (single dose, 20 mg/kg i.p.), MTX + HES group, and MTX + HES-BS group (70 mg/kg HES and HES-BS were orally administered 2 days before and 14 days following the MTX injection) [30]. The Institutional Animal Care and Use Committee (IACUC) of Beni-Suef University in Egypt provided the recommendations for this research (BSU/2023/5/23).

#### Samples Preparation

By the end of the experiment, the animals were given adequate anesthesia via intraperitoneal injection at a dosage of 0.2 ml/100 g.b.wt. and consisting of a mixture of both ketamine (90 mg/kg body weight) and xylazine (5 mg/kg body weight) in a 1:1 ml ratio. All animals were terminated ethically. For serum samples, Blood collected from the jugular vein of the rats and permitted to clot at the ambient temperature and rotated rapidly at a speed of 3000 revolutions per minute for 30 min using a centrifuge. Then, the transparent, non-hemolyzed sera were promptly extracted and split into three sections for each animal. The sera were subsequently stored at -20 °C for further analysis.

#### Processing of renal tissue for oxidative stress and molecular biomarker assessment

The kidneys were promptly removed following the dissection of the euthanized animals. The renal tissue samples were rinsed with a PBS (pH 7.4) that included 0.2 mg/ml of heparin to eliminate red blood cells. Next, the tissue was crushed into a uniform mixture using 5–10 ml of a cold solution of 100 mM potassium phosphate, pH 7, and 2 mM EDTA / gram of tissue. The mixture was spun at 4,000 rpm at a temperature of 4 °C for 15 min. Then, the resultant supernatant was obtained and kept at -80 degrees Celsius. This frozen sample would be used to examine oxidative stress indicators. Another part of the kidney was kept in RNAlater at -80 °C and used for molecular studies.

### Renal function tests

Reagent kits purchased from Spinreact (Spain) were utilized to analyze serum creatinine, urea, and uric acid serum levels. The procedures employed by Young (1995), Kaplan (1984), and Fossati et al. (1980), respectively, were applied. eGFR was determined by utilizing the CKD-EPI creatinine equation [31]. Na and K were measured using an automated analyzer (EasyLyte^®^ analyzer).

### Oxidative stress markers

MDA was evaluated using the methodology established by Ohkawa et al. [32]. NO level was evaluated using the method of Montgomery and Dymock [33]. GSH concentration was evaluated according to Beutler *and* Kelly [34]. The investigation of SOD was conducted using the methodology described by Nishikimi et al. [35]. Moreover, GPx was determined using the method described by Paglia and Valentine [36].

### Molecular investigations

#### Total RNA extraction

Cellixizol reagent was employed to isolate the entire RNA from frozen kidney samples (cellixiza, Germany). The monophasic solution of phenol and guanidine isothiocyanate improves the single-step RNA isolation procedure and yields high Total RNA. Isolated RNA was measured using a nano-drop UV-Vis spectrophotometer (Nano-Drop 2000 by ThermoFisher) to determine RNA purification statements. Subsequently, it is prepared for downstream applications, including cDNA synthesis.

#### The quantitative real-time PCR

The High-performance cDNA Reverse Transcription Kit (Cellixiza, Germany) synthesized cDNA following the manufacturer’s instructions. All of the primers demonstrated a melting temperature ranging from 59 to 61 °C, and they were prepared by Eurofins Biomnis, France. All RT-PCR procedures used 20 µl of SYBR Green master mix (cellixiza, Germany) with cDNA. Subsequently, qRT-PCR was performed under the manufacturer’s specifications (Applied Biosystems S/N: 2710004586). This thermal profile was used: 5 min at 95 °C, 40 cycles of 30 s at 95 °C, then 30 s (57–60) °C and 72 °C respectively. The last stage was kept at 72 °C for 15 min.

After PCR amplification, the 2ˆ (–delta delta CT) ΔΔCt was calculated by subtracting the *β-actin* Ct from each sample Ct [37]. *β-actin* is utilized as a reference gene. The primer sequences for qRT-PCR are presented in Table 1.

**Table 1:** Primer sequences for real-time PCR

Gene	Forward	Reverse
*Nrf2*	CATTTGTAGATGACCATGAGTCGC reference number: 11-3062-64/711 0	ATCAGGGGTGGTGAAGACTG reference number: 11-3062-65/711 0
*Keap1*	CTTCGGGGAGGAGGAGTTCT reference number: 11-3062-66/711 0	CGTTCAGATCATCGCGGCTG reference number: 11-3062-67/711 0
*Bcl2*	CCGGGAGATCGTGATGAAGT reference number: 11-3062-60/711 0	ATCCCAGCCTCCGTTATCCT reference number: 11-3062-61/711 0
*Bax*	GTGGTTGCCCTCTTCTACTTTG reference number: 11-3062-62/711 0	CACAAAGATGGTCACTGTCTGC reference number: 11-3062-63/711 0
*β-actin*	TGACGAGATGCAGAAGGAGA reference number: 11-3062-74/711 0	TAGAGCCACCAATCCACACA reference number: 11-3062-75/711 0

### Histopathological examination

The renal tissue was removed and stored in 10% formalin at ambient temperature for 72 h. Subsequently, the product was dehydrated using a variety of alcohol and water mixtures and cleansed with xylene. The samples were placed in a paraffin core. Subsequently, sections of tissues were deparaffinized and dyed with Hematoxylin and Eosin (H&E) before being examined under a light microscope (Olympus CX41 microscope, Olympus, Japan) [38]. The sections were cut to 4–5 μm thickness using a microtome.

### Statistical analysis

The statistical study was performed utilizing the Statistical Package for Social Sciences (IBM SPSS for WINDOWS 8, version 22, SPSS Inc., Chicago). Before analysis, the data distribution was evaluated with the Shapiro-Wilk test for normality and Levene’s test for homogeneity of variances. Given that the criteria for normal distribution and homogeneity of variances were satisfied (*p* > 0.05), parametric tests were utilized. To ascertain the statistically significant differences between groups, the data underwent a one-way analysis of variance and Duncan’s Multiple Range Test. Results were presented as mean ± standard error (SE) with P values < 0.05 indicating significance.

## Results and discussion

### Characterization of hesperidin-bilosomes nano formula

#### Zeta sizer and potential of HES-BS

**Fig. 1:**
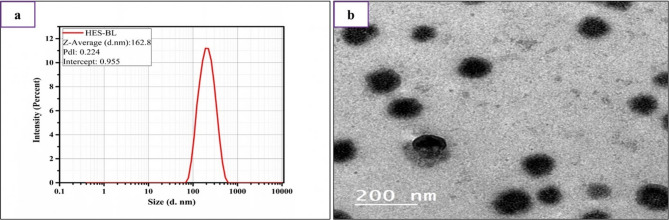
(**a**) Particle size, Polydispersity index (PDI) of hesperidin–bilosomes, and (**b**) transmission electron microscope micrographs of HES-BS

Particle size is an important measurement during the preparation of nano formulas due to its impact on nanoparticle stability, release behavior, and biodistribution [39]. In our study, the reported particle size of the HES-BS nano formula was 162 nm. This finding proves that the synthetic nano formula falls below 1000 nm on the nanometric scale, as shown in Fig. 1a.

Our prepared formula can improve how hesperidin is absorbed and processed in the body by enhancing its oral bioavailability. This is achieved by reducing the particle size to less than 500 nm, which promotes the absorption of hesperidin by enterocyte uptake [40]. Moreover, it has been shown that polymeric nanoparticles smaller than 500 nm can cross the M cells in the gut and be absorbed by the lymphatic system. This phenomenon allows them to bypass initial liver metabolism and enhance their bioavailability [41]. Furthermore, the increase in the SDC ratio resulted in a notable reduction in surface tension and interfacial tension within vesicle bilayers, leading to enhanced flexibility of the vesicle membrane [42]. In turn, this increased the space between vesicle bilayers [43].

On the other hand, the zeta potential measurement indicates particle surface charge [44]. Moreover, it is an essential physicochemical indicator of the stability of nanosuspensions. The data we collected showed that the zeta potential of the HES-BS nanoformula was − 21.6 mV. This indicates that the formula had sufficient charge to avoid particle aggregation and confirms the excellent physical stability of the bilosome formula. Generally, the electrostatic repulsion between particles transported by high zeta potential values is vital for minimizing the aggregation of nanoparticles [45]. The overall charge of the hesperidin bilosome structure was regulated by adjusting the molar ratio between the anionic SDC and the neutral SPC. In other words, if the concentration of bile salts is increased, vesicles with significantly large negative ZP values will be created [46–48].

Our data showed that the polydispersity index (PDI) of the prepared HES-BS nanoformula was 0.224, reflecting the good size uniformity of the nanoformula (Fig. 1a). Furthermore, PDI values below 0.5 imply that the nanoparticle (NP) dispersion is homogeneous and has a restricted size distribution [49]. Moreover, the results showed no significant changes in particle size, polydispersity index (PDI), zeta potential, or encapsulation efficiency, after 3 months of storage at 4℃, indicating short-term physical stability of the prepared HES-BS formulation. Furthermore, controlling particle size and surface charge is significant; therefore, our prepared nanoformula could collectively increase the therapeutic efficacy of hesperidin and maximize its predictable in vivo outcomes.

#### Transmission Electron microscope (TEM)

Transmission Electron Microscopy is a crucial system for validating the findings obtained from the Malvern particle size analyzer [50]. It was utilized to confirm the development of BS vesicles and observe their structure [20]. Figure 1b demonstrated that the HES-BS nanoformula displayed a sphere-shaped morphology, with a particle size of approximately 162 nm, which agreed with that determined by the zeta sizer. The particles also showed no obvious aggregations and had smoothly free surfaces, which confirmed their stability. The TEM micrographs from several studies demonstrated that BS vesicles had a spherical or near-spherical morphology. The shape and properties of nanoparticles (NPs) significantly impact interactions with the biological system, enterocyte uptake, and distribution within the body [51]. Furthermore, reports indicate that spherical nanoparticles are taken up by cells more effectively than their rod-shaped counterparts. In contrast to rod-shaped nanoparticles, spherical ones are less influenced by the cytoskeleton and are ultimately taken up by cells at a higher speed [51].

#### Entrapment efficiency percentage (EE %)

Bilosomes have the potential to be used as an oral delivery system due to their ability to encapsulate huge quantities of medication. In our study, the EE% of the HES-BS nanoformula was 89.1%, indicating that the drug loading had been performed effectively. The drug’s lipophilicity was key to entrapment within the bilosomal lipid phase [52]. The current formula exhibited a high EE percentage, likely attributed to the drug’s lipophilic properties and the affinity of BS-vesicles for its lipid bilayer areas.

Furthermore, it was noted that the encapsulation of various medications increased as the concentration of bile salts in the vesicles increased. Bile salts, which have surface-active features, increase the flexibility and ability of the medicine to dissolve in the membrane [53]. Additionally, when the quantity of bile salts rises, the solubility of the medication in the dispersion medium also increases [54]. Moreover, the augmentation of the lecithin concentration resulted in a notable rise in the encapsulation efficiency percentage of vesicles. That may be attributed to the production of a greater number of particles for trapping the drug [55]. Meanwhile, loading capacity and regulated release of medications often depend on the drug’s chemical properties and its interaction with the carriers [56].

### FTIR spectra analysis

**Fig. 2:**
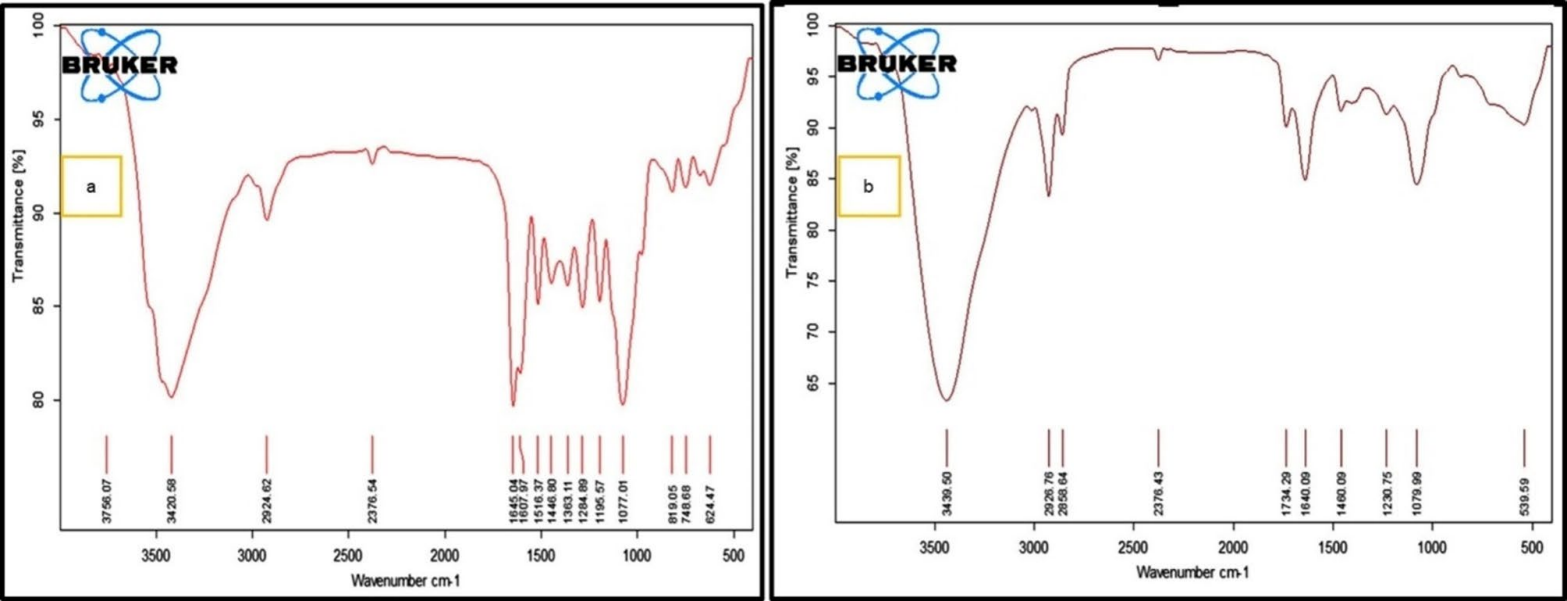
Fourier-transform infrared spectroscopy (FTIR) spectra represent the FTIR Spectrum of hesperidin and HES–BS nano formula

FTIR analysis is necessary to determine HES and HES-BS functional groups. FTIR analysis of pure HES and nanoformulated hesperidin were measured in the 4000–400 cm^− 1^ spectral range and shown in Fig. 2. Pure hesperidin exhibited distinct bands corresponding to various functional groups as represented in Fig. 2a., such as 3420.55, 2924.5, 1645.42, 1516.38, and 1076.99 cm^− 1^. These bands result from stretching vibrations of O–H, C–H, C = O, C = C, and C–O, respectively. Moreover, the measured peak matched the literature data [57].

The spectra of both HES and HES-BS exhibit almost identical characteristic peaks, with only slight variations due to the presence of the lecithin and SDC spectra, as shown in Fig. 2b. The findings suggested that there was no interaction between the components of bilosomes and HES and that HES was successfully encapsulated inside the bilosomes.

### DSC study

**Fig. 3:**
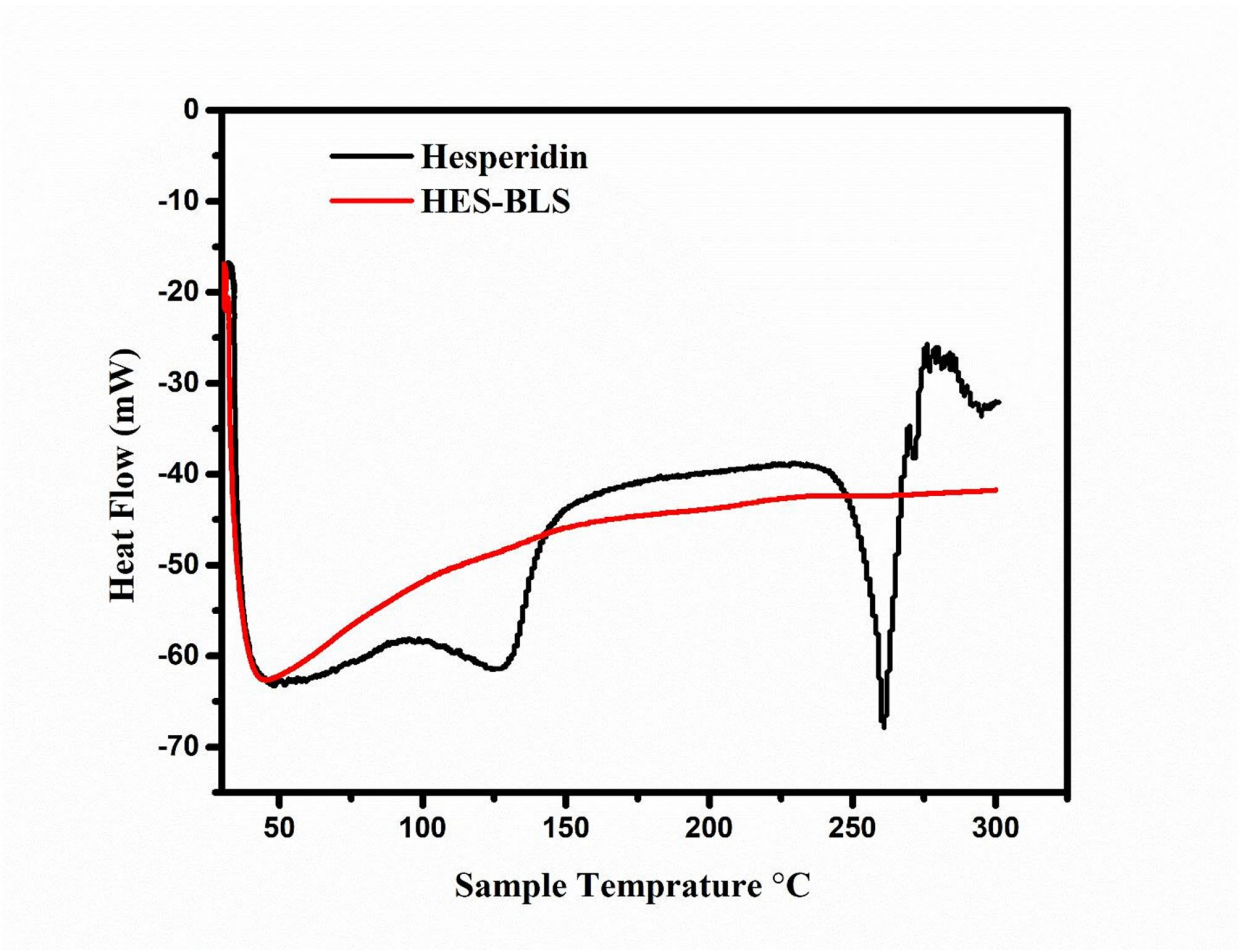
DSC curves of hesperidin and HES–BS nanoformula

A thermal analytical method called differential scanning calorimetry was used to assess the HES sample’s physical properties, purity, and interactions with excipients. The thermograms of crude HES and HES-BS nanoformula are shown in Fig. 3. The DSC thermogram of pure HES exhibited a characteristic endothermic peak at 260.99 ˚C (Fig. 3), corresponding to HES melting and proving its crystalline state which agreed with the existing literature [58]. In addition, the thermogram of HES-BS displayed an endothermic peak at temperatures of 216 and 272 °C (Fig. 3). Notably, the lack of the endothermic melting peak of HES suggests that HES was completely enclosed inside the nanoparticles in an amorphous form. Consequently, the encapsulated HES exhibited distinct thermal characteristics compared to pure HES. The hydroxyl (-OH) group of hesperidin and the polar portion of the lipid molecule establish hydrogen bonds, facilitating interaction between the lipid and HES. As the temperature increases, the interaction between HES and lipids forms a complex with a melting point different from that of either of the separate components.

### In-vitro drug release (DR) studies

**Fig. 4:**
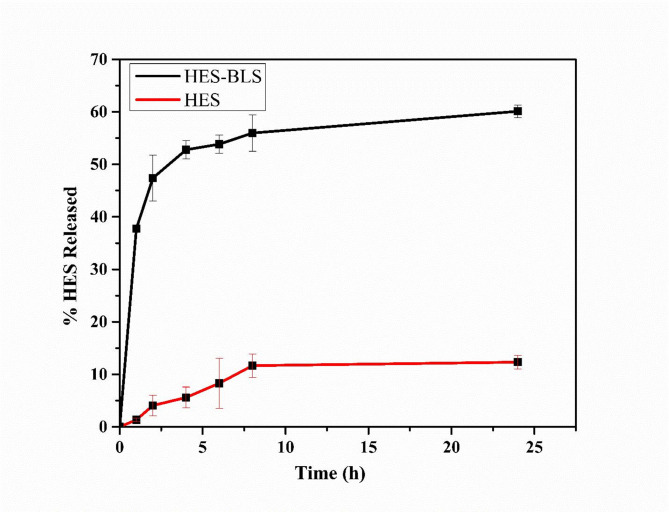
Comparative in vitro drug release of HES-loaded BS and HES suspension

Figure 4 displayed the cumulative drug release of the HES-BS nanoformula, revealing a two-phase sequence of HES release from the bilosome. This pattern consisted of an initial fast release of approximately 55.96% of the HES within the first 8 h, followed by a sustained and gradual release of about 60.1% of the HES over the subsequent 24 h. On the contrary, the crude HES gradually released around 12.32% during 24 h.

Drug release refers to the movement of drug solutes from their original location within a material system to the medium in which they are released [59]. The release study of BS-vesicles was assessed by employing the dynamic dialysis approach to avoid vesicle leakage into the outside release media [60]. The process is influenced by several intricate elements, including the drug solutes’ physicochemical properties, the material system’s features, the release environment, and the interactions between these factors [61]. The primary factors that propel the transport of medication solutes include diffusion and expansion combined with system material degradation [62].

**Fig. 5:**
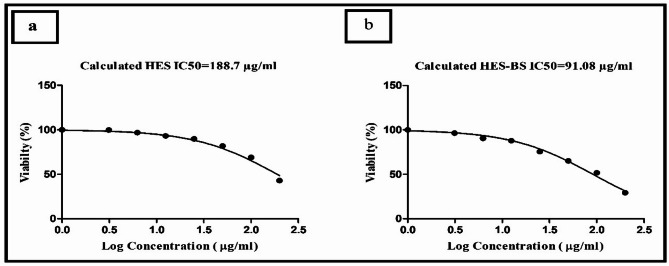
Treatment of Vero Cells with HES-BS effectively reduces the IC_50_ value from 188.7 µg /ml (**a**) to 91.08 µg/ml (**b**) using MTT assay

In the present study, the formulation of HES-BS markedly improves the apparent permeability of HES, which could be attributed to the internalization into BS vesicles, active uptake in addition to its lipidic fusion as also occurred with solid lipid nanoparticles, which was conveyed previously [63, 64]. So, the observed first burst phase is attributed to the release of HES from the surface of the outer layer of the bilosomal system. Meanwhile, the second prolonged phase occurred due to the strong attraction between HES (lipophilic compound) and the inner lipophilic layer of the bilosomal system. This biphasic release behavior of HES nanoparticles was similarly reported in previous research [65]. On the contrary, crude HES is water-insoluble and poorly transported via the dialysis membrane that matched with previous study of Alam et al. (2023) [66]. Hence, the sustained release of HES-BS, with its specific size, slightly negative charge, and reduced IC50 (Fig. 5), could directly maximize its in vivo therapeutic efficacy.

### Biochemical markers

#### Leverage of HES-BS on renal function tests

Concerning the kidney function panel (Table 2), our data showed that MTX induced a renal injury with a significant increase in urea, uric acid, creatinine, and potassium (K) serum levels (*P* < 0.001). On the contrary, the eGFR and sodium (Na) concentrations substantially reduced in the MTX-group compared to the control group. The present data agreed with the finding of Al-Abkal et al., who stated that MTX precipitated in the renal tubules and resulted in a notable decrease in eGFR levels. At the same time, urea, creatinine, and uric acid serum indicators were significantly increased, a feature of renal disorders and nephrotoxicity [67].

**Table 2:** Effects of HES and HES-BS on kidney function tests

Groups	Urea (mg/dL)	Creatinine (mg/dL)	eGFR (mL/min)	Uric acid (mg/dL)	Sodium (mmol/L)	Potassium (mmol/L)
Normal Control	31.00 ± 0.68^d^	0.35 ± 0.02^d^	188.50 ± 3.80^a^	1.63 ± 0.27^d^	147.00 ± 1.09^ad^	4.75 ± 0.07 ^b c^
MTX-Group	54.83 ± 1.35^a^	1.32 ± 0.03^a^	89.33 ± 2.55 ^d^	3.72 ± 0.12^a^	133.50 ± 1.28^cd^	5.01 ± 0.09 ^a^
Bilosome	47.33 ± 3.17^b^	1.28 ± 0.04^a^	92.67 ± 3.44 ^d^	3.85 ± 0.08^a^	132.16 ± 0.94^d^	4.90 ± 0.07 ^a b^
Hesperidine	39.67 ± 1.41^c^	0.87 ± 0.08^b^	138.17 ± 7.35^c^	3.05 ± 0.17^b^	135.66 ± 1.11^c^	4.46 ± 0.11 ^d^
Bilosome-Hesperidine	33.67 ± 0.49^d^	0.53 ± 0.03^c^	166.33 ± 3.27^b^	2.15 ± 0.1^c^	141.83 ± 1.19^b^	4.60 ± 0.07^cd^
P -value	0.000	0.000	0.000	0.000	0.000	0.001

Data are expressed as mean ± SE, means which share the same superscript symbol(s) are not significantly different, *P* < 0.001

The current data demonstrates that MTX has a nephrotoxic impact, evidenced by elevated renal function and electrolyte imbalances characterized by hyponatremia and hyperkalemia after the injection of MTX. Additionally, MTX directly affects renal tubular epithelial cells, decreasing epithelial transport activity and tubular sodium reabsorption, which manifests as hyponatremia [68]. In addition, MTX has the potential to create solid particles inside the tubular lumen, leading to obstructions in the tubules and subsequently lowering the excretion of potassium. This reduction in excretion is followed by the release of intracellular potassium into circulation [69]. Renal tubular cell injury will disrupt the process of regenerating tubular cells and hinder their normal functioning, decreasing the eGFR level. Moreover, these cells may detach and enter the tubular lumen, causing a localized decrease in the flow of tubular fluid and exacerbating the formation of MTX crystals in particular tubules [70].

Our current data indicated that administering HES and HES-BS drastically decreased urea, uric acid, creatinine, and potassium (K) levels in the blood. In contrast, the levels of eGFR and Na showed a substantial rise in the treated groups contrasted with the MTX - group, as shown in Table 1. Furthermore, the group that received treatment with HES-BS demonstrated a noteworthy improvement in renal function compared to the MTX-HES group. Consistent with our findings, Caglayan et al. revealed that HES effectively safeguarded the kidneys by reducing serum urea and creatinine levels [71]. Furthermore, Alsawaf et al. demonstrated that the administration of HES improved the changes in serum sodium, serum and urine creatinine, blood urea nitrogen, and relative kidney mass among all treated rats [72].

The nephroprotective action of HES is attributed to its anti-inflammatory and antioxidant properties, which enable it to heal damaged tissue and reduce tubular cell vascular abrasions induced through toxic substances [73]. In addition, HES-BS nanoformula increases HES’s bioavailability, maximizing its antioxidant and anti-inflammatory potential effect against nephrotoxicity disorders compared to crude HES. Consistent with our findings, Elkomy et al. observed that BS vesicles had a significant capability to traverse the cellular membrane and enhance penetration activity, making the oral delivery of the formulated drug more effective [15].

### Effect of HES-BS on oxidative stress markers

**Fig. 6:**
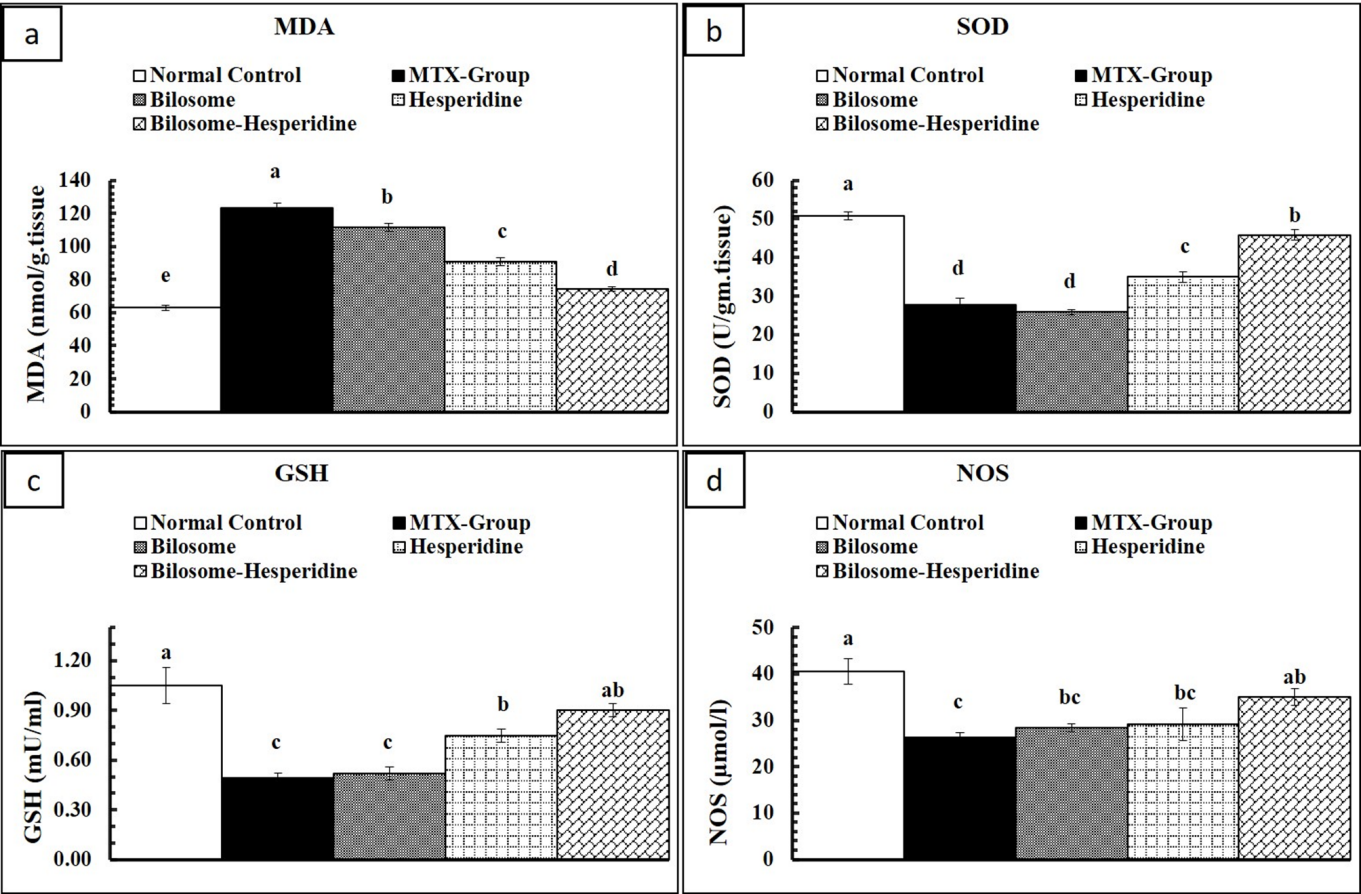
Effects of HES and HES-BS on oxidative stress indicators: (**a**) MDA, (**b**) SOD, (**c**) GSH, and (**d**) NOS. Data are expressed as mean ± SE, means which share the same superscript symbol(s) are not significantly different, *P* < 0.001

Figure 6 showed that MTX administration significantly decreased glutathione and superoxide dismutase, while malondialdehyde and nitric oxide were notably reduced compared to the control group. Previous results indicated that MTX treatment induced a renal cytotoxic effect and led to a notable reduction in GSH concentration, while MDA and NO concentrations exhibited a considerable increase [70, 74]. In addition, MTX can destroy cells by generating excessive reactive oxygen species (ROS) through the mitochondria. As a result, the efficiency of antioxidant enzymes is reduced, causing oxidative damage to crucial subcellular macromolecules and ultimately resulting in increased lipid and protein oxidation levels, leading to renal apoptosis [29, 75, 76].

Furthermore, nitric oxide may enhance the impact of reactive oxygen species (ROS) by interacting with superoxide radicals, intensifying peroxynitrite production. Peroxynitrite is a potent oxidizing agent that can further contribute to the degradation of lipids and proteins [77]. In addition, Craven et al. (1992) found that oxidative stress might trigger the secretion of several vasoactive substances, leading to renal vasoconstriction and a decrease in the glomerular filtration rate [78]. This may explain the previously noted decline in renal function caused by MTX.

Figure 6 showed that treatment with HES and HES-BS nanoformulas significantly increased the levels of GSH and SOD in all treated groups compared with the MTX- group. On the contrary, MDA and NO levels were notably decreased in the MTX-HES and HES-BS groups when compared with the MTX- group.

Moreover, the HES-BS nanoformula exhibited the highest potency, restoring antioxidant activities similar to the control group’s. Furthermore, the BS vesicles increased the pharmacokinetic activity of HES, enhancing its biodistribution, antioxidant, and anti-inflammatory properties. Oxidative stress and inflammation are the fundamental processes responsible for developing kidney damage caused by MTX. Therefore, using substances that possess antioxidant and anti-inflammatory characteristics might be a promising strategy for improving the effectiveness of MTX in therapy settings. Hesperidin fights free radicals and reactive oxygen species to protect cells from oxidative stress [79]. Moreover, HES effectively reduced lipid peroxidation in kidney tissue, as indicated by the reduction in MDA concentration [80]. This was followed by a rise in GSH content and higher activity of SOD, as reported by Abd-Eldayem et al. [80]. These observed outcomes may be ascribed to the possible antioxidant properties of HES, which have also been shown in many prior investigations [81–83].

### Effect of HES-BS on gene expression

To understand the molecular concept behind the MTX’s behavior regarding nephrotoxicity and the molecular effect of the prepared HES-BS nano formula against its toxic activity, we study two mechanistic pathways of *Nrf2*/*Keap1* and the *Bax*/*Bcl2* ratio.

**Fig. 7:**
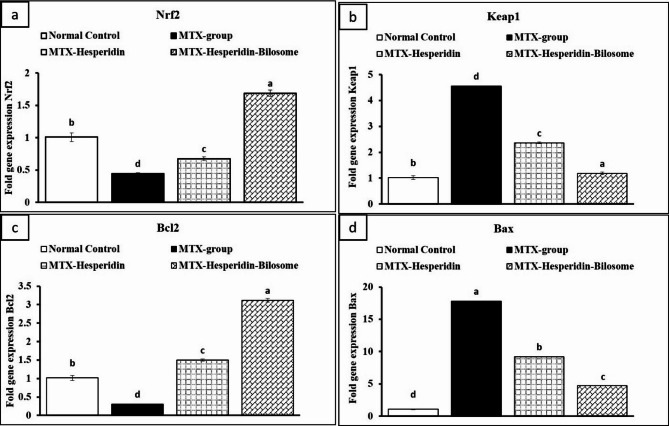
Effects of HES and HES-BS on gene expression of (**a**) Nrf2, (**b**) Keap1, (**c**) Bcl2, and (**d**) Bax in the kidney tissue of rats. Data are expressed as mean ± SE, means which share the same superscript symbol(s) are not significantly different, *P* < 0.001

Our study demonstrated that MTX significantly reduced Nrf2 mRNA expression in renal tissues. However, the expression of Keap1 was considerably higher in the MTX-group as compared to the normal group, as seen in Fig. 7. The current research corroborated the results of Sayed et al. who detected a substantial decrease in the expression of Nrf2 in the group treated with MTX. Additionally, they noted a large rise in the expression of the Keap1 gene after methotrexate induction [84]. Nrf2 possesses anti-inflammatory functions and is crucial in safeguarding the kidney from many illnesses [85, 86]. Also, Nrf2 is regarded as a potential and novel target for therapeutic drugs utilized in curing chemical-induced chronic damage to renal cells. The cytoprotective effects of Nrf2 activation entail the removal of toxins and ROS, which are very significant in renal toxicity [87]. While mild oxidative stress triggers the activation of Nrf2, severe and prolonged production of ROS may inhibit Nrf2 signaling in the kidney tissue of MTX-treated rats [85]. The reduced activity of the Nrf2 pathway and the increased activity of the Keap1 pathway directly result from the continuous manufacture of ROS caused by MTX.

The current data indicate that the administration of free HES and HES-BS nano formulas led to a notable rise in Nrf2 expression while simultaneously suppressing Keap1 gene expression in all treated groups compared to the MTX group. Moreover, the findings demonstrated the remarkable effectiveness of the HES-BS nanoformula. The results elucidated the strong antioxidant capabilities of HES-BS by influencing the activity of Nrf2, which controls internal natural defense mechanisms against oxidative stress. The reduction in Keap1 levels allows for greater availability of Nrf2, resulting in enhanced protection [88]. Nevertheless, in response to oxidative stress, stressors directly attaching to reactive cysteine residues modify Keap1’s conformation, inhibiting Nrf2 ubiquitination. Instead of being released through Keap1, Nrf2 covers all of its binding sites, enabling freshly produced Nrf2 to evade Keap1, translocate to the nucleus, and increase the expression of cytoprotective genes [89].

**Fig. 8:**
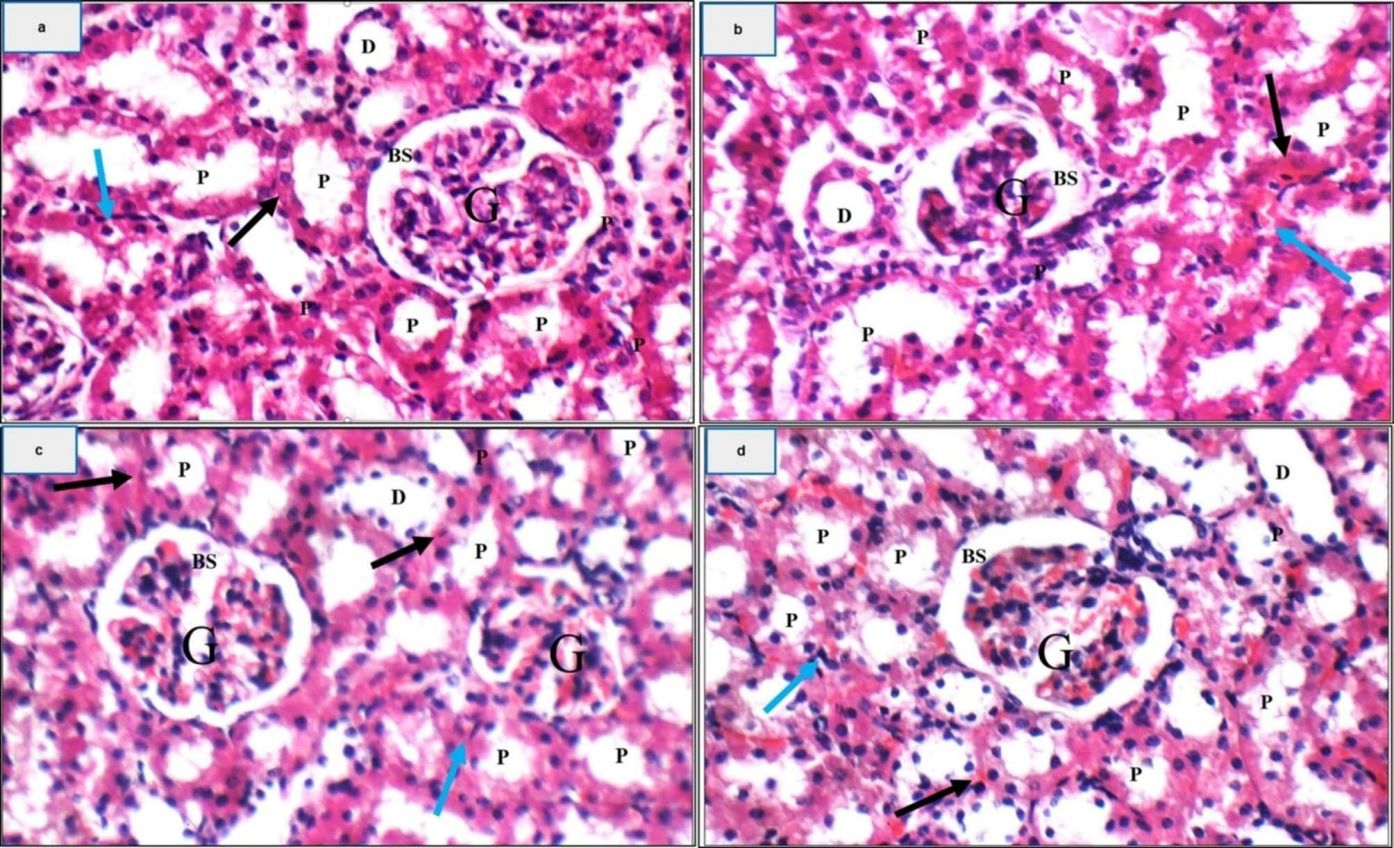
H&E X 400 of rat kidneys from (**a**) Negative control: high power view showing average glomeruli (G) with average Bowman’s spaces (BS), proximal tubules (P) with average epithelial lining (black arrow), and average interstitial blood vessels (blue arrow); (**b**) MTX group: high power view showing small-sized glomeruli (G) with widened Bowman’s spaces (BS), proximal tubules (P) with scattered apoptotic epithelial lining (black arrow), and average interstitial blood vessels (blue arrow); (**c**) MTX + HES: high power view showing specific amelioration but the proximal tubules (P) with scattered apoptotic epithelial lining (black arrow) (**d**) MTX + HES-BS: showing normal histopathological features for all parameters

The findings of our study demonstrated a marked decrease in Bcl2 gene expression, with a notable elevation in the expression of the Bax gene in kidney tissue following treatment with MTX, as shown in Fig. 8. This indicates a shift toward a higher Bax/Bcl2 ratio. The data we obtained supported the results of Abdelkader et al., who detected that treatment with MTX induced renal apoptosis by upregulating the expression of Bax in renal tissue while drastically downregulating the expression of the anti-apoptotic protein Bcl2 [90]. The stimulation of apoptosis-related signaling pathways is widely believed to be connected with the direct lethal effects of MTX [91, 92]. Therefore, induction of apoptosis could be considered among the main contributing factors of MTX-induced nephrotoxicity.

In contrast, all other treated groups showed a considerable elevation in Bcl2 expression and a drop in Bax levels in kidney tissue after receiving HES or HES-BS compared to the MTX-treated group. As observed in Fig. 8, the HES-BS formula exerted the greatest influence. Treatment with HES and HES-BS nanoformula reduces kidney damage and inhibits the *Bax/Bcl2* signaling pathway by activating the anti-apoptotic gene *Bcl2*, thereby enhancing its potential to protect the kidneys. Additionally, Aggarwal et al. revealed that administering HES could inhibit programmed cell death by regulating the levels of proteins involved in developing apoptosis and anti-apoptosis [93]. Moreover, the *Bcl2* gene can impede a diverse range of apoptotic signals and hinder the release of cytochrome C, which is regarded as a crucial element in activating caspases that facilitate apoptosis [94]. In contrast, Bax was the first protein to share two highly conserved sequences with *Bcl2*. *Bax* homodimers induce apoptosis when overproduced and dimerized with *Bcl2* or itself. In excess *Bcl2*, homodimers prevail and protect cells from death [95]. Moreover, Sharifi et al. also found that the relationship between Bax and Bcl2 expression is a cell death adjustment that regulates cell survival in response to apoptotic stimuli. Elevated Bax/Bcl-2 ratios diminish cellular resistance to apoptotic stimuli, increasing cell death and decreasing tumor prevalence [96]. Also, Erboga et al. stated that an overabundance of apoptosis or its improper regulation can result in nephrotoxicity [97]. Furthermore, the administration of HES-BS before MTX demonstrated a dual capacity to reduce oxidative damage and inflammatory processes, as evidenced by the decreased levels of Bax and the increased expression of the anti-apoptotic protein Bcl2.

### HES-BS mitigates the histopathological alterations in the kidney caused by MTX

Figure 8 displayed the renal tissue of the normal control group, which exhibited normal histology features. On the contrary, MTX-treated rats exhibited significant histological abnormalities as a result of MTX poisoning, such as degenerative alterations in the glomeruli and renal tubules. Semi-quantitative histopathological scoring supported the qualitative findings, showing marked renal tissue damage in the MTX group and near-normal architecture in the Bilosome-Hesperidin group (Supplementary Table S1). The kidney exhibited remarkable damage to the renal capsule, with small glomeruli showing expanded Bowman’s spaces. The proximal tubules displayed disseminated apoptotic epithelial lining, while the distal tubules appeared average. The interstitium and kidney medulla exposed average characteristics, with collecting tubules displaying scattered apoptotic epithelial lining. Our findings were consistent with Ahmed, Zaki, and Nabil’s statement that methotrexate had a deleterious impact on tubular epithelium cells owing to its toxic harm. They observed dilations of the tubules with the buildup of casts, cellular fragments, and localized intrusion of inflammatory cells within the peritubular area. Elevated levels of MTX cause the buildup of MTX crystals inside the nephron with an enlargement of the kidney tubules, leading to nephrotoxicity. Also, the histologic changes could be related to the creation of ROS and free radicals. In the hesperidin-treated group, the kidney showed average amelioration of all recorded parameters but with scattered apoptotic epithelial lining. Where, HES possesses cytoprotective, antioxidant, and antiapoptotic effects. Treatment with HES significantly reduced tubular necrosis by enhancing the cellular antioxidants. It is interesting to note that hesperidin improved the pathological abnormalities, preventing the cellular infiltration inside renal tissue generated by MTX and retaining the glomerular, tubular, and interstitial kidney structure, as previously described [80]. The kidney in the HES-BS nanoformula kept all histopathological characteristics in the normal state. This investigation revealed that HES-BS augmented the effects of hesperidin and improved MTX-induced pathological changes better than HES alone.

## Conclusion

The present study indicated that hesperidin was successfully encapsulated within a bilosome in a well-characterized form, measuring 167 nm in size and having an entrapment efficacy of 89.1%. The HES-BS nanoformula significantly protects against nephrotoxicity induced by MTX in rats. The prepared formula effectively relieved the altered biochemical kidney function and ameliorate oxidative stress by increasing antioxidant parameters. Moreover, the prepared formula increased the anti-apoptotic properties of hesperidin by alleviating BAX/BCL2 and Nrf2 /Keap1 pathways. Furthermore, the induced histopathological changes were also assuaged. Therefore, hesperidin’s antioxidant, anti-inflammatory, and anti-apoptotic properties were effectively enhanced using the prepared HES-BS nanoformula, which could be considered an effective remedy for nephrotoxicity and various clinical conditions.

## Electronic supplementary material

Below is the link to the electronic supplementary material.

Supplementary Material 1

## Data Availability

No datasets were generated or analysed during the current study.
