# Supplementary material for: Hesperidin loaded bilosomes mitigate the nephrotoxicity induced by methotrexate; biochemical and molecular in vivo investigations
Source: BMC Nephrol. 2025 Jul 21;26:404. doi: 10.1186/s12882-025-04328-4 (PMC12278527; doi:10.1186/s12882-025-04328-4)
Supplement: Supplementary file 1 — Supplementary Material 1 [file 12882_2025_4328_MOESM1_ESM.docx]

**Supplementary Table S1**

**Table S1. Semi-quantitative histopathological scoring of kidney sections from different experimental groups, based on changes in glomeruli, Bowman’s space, tubules, interstitium, and medulla.**

|  | **G** | **BS** | **Tubules** | | | **Interstitium** | **Medulla** |
| --- | --- | --- | --- | --- | --- | --- | --- |
|  |  |  | **Lining** | **Brush border** | **Lumen** |  |  |
| **Normal Control** | 0 | 0 | 0 | 0 | 0 | 0 | 0 |
| **MTX-Group** | + | + | + | + | 0 | 0 | + |
| **Hesperidine** | 0 | 0 | + | 0 | 0 | 0 | 0 |
| **Bilosome-Hesperidine** | 0 | 0 | 0 | 0 | 0 | 0 | 0 |

- **Glomeruli (G):**

0: Average +: Small-sized ++: atrophied/hypercellular

- **Bowman’s spaces (BS):**

0: Average +: Widened/dilated ++: Obliterated

- **Tubules**
- **Lining:**

0: Average +: Edematous/scattered apoptotic ++: Necrotic/markedly apoptotic

- **Brush border:**

0: Preserved +: Partial loss ++: Complete loss

- **Lumen:**

0: Free +: Few/scattered casts ++: Marked Intra-tubular casts

- **Interstitium:**

0: Average +: Dilated/congested BV ++: Markedly dilated BV/interstitial inflammatory infiltrate

- **Medulla:**

0:Average +: Mildly congested capillaries /scattered apoptosis ++: Markedly congested capillaries /hyaline casts
